# Diagnostic tests for urinary tract infections and antimicrobial resistance—a reality check between diagnostic innovation and implementation: a critical narrative review

**DOI:** 10.3389/fcimb.2026.1784139

**Published:** 2026-05-15

**Authors:** Vathsala Mohan, Konstantinos Mitsakakis

**Affiliations:** 1 Commonwealth Scientific and Industrial Research Organisation (CSIRO), Health and Biosecurity, Westmead, NSW, Australia; 2 Hahn-Schickard, Freiburg, Germany; 3 Laboratory for MEMS Applications, IMTEK–Department of Microsystems Engineering, University of Freiburg, Freiburg, Germany

**Keywords:** artificial intelligence and UTIs, biomarkers, epidemiology, novel sample collection logistics and rapid diagnostics, point of care, urinary tract infection, UTI diagnosis, UTI management

## Abstract

Urinary tract infections (UTIs) are a significant health concern worldwide, leading to substantial health and economic burden and contributing to the rise of antimicrobial resistance (AMR). Although there has been significant advancement in diagnostic technologies starting from conventional dipstick urinalysis to molecular assays and artificial intelligence (AI)-supported platforms, their clinical utility and impact still remain unclear. This is fundamentally due to the systemic barriers that hinder the implementation including cost, policy, and limitations to integrate into frontline healthcare and adoption by insurance agencies. This review focuses on critically reviewing UTI epidemiology, current and emerging diagnostics, and their significance in antimicrobial stewardship. We emphasise that bridging the gap between diagnostic innovation and clinical practice is essential to achieving meaningful milestones in AMR reduction. We are primarily arguing that addressing the policy inertia to diagnostic deployment with stewardship strategies is a crucial step for improving patient healthcare outcomes and preserving antimicrobial efficacy.

## Introduction

Urinary tract infections (UTIs) being one of the most common clinical conditions pose significant health and economic concerns worldwide affecting children, men, women, and elderly populations. Despite the negative and long-term unpleasant impacts caused by UTIs experienced, the diagnosis and management of UTIs remain inconsistent and often empirically driven in both developed and developing countries (Öztürk and Murt, 2020; Brubaker et al., 2023). This in turn has led to inappropriate use of antimicrobials, thereby making UTIs a significant driver for the emergence of antimicrobial resistance (AMR) in both community and healthcare settings.

It is crucial to diagnose UTIs in a timely manner but also to monitor accurately during treatment to avoid complications, to facilitate appropriate treatment and improve clinical symptoms and support appropriate management of UTI reducing the risk of antimicrobial resistance (NICE, 2023). Even though a wide range of diagnostic tools are available including manual and automated dipstick assays, urine cultures, and more advanced molecular and biomarker-based platforms, most of the advanced diagnostic tools are under-utilised and sometimes misapplied, particularly in frontline or primary care (Australian Commission on Safety and Quality in Healthcare, 2021; Rowe et al., 2022; Bono et al., 2023; WHO, 2023).

Often, clinical decisions are made in the absence of definitive microbiological confirmation where guidelines on the application of diagnostic testing differ among countries, and in most instances, treatment is initiated even before pathogen identification, which leads to unguided antibiotic use exposing the individual to an increased risk of resistance development (Crnich et al., 2015; Rowe et al., 2022; Bono et al., 2023). For example, UTIs are treated more empirically using third-generation cephalosporins for uncomplicated cystitis and based on the local antibiogram data (Miller et al., 2018; Benning et al., 2023; Kusin et al., 2023). Some countries provide guidelines for the treatment of uncomplicated cystitis such as the Infectious Diseases Society of America (IDSA). It is reported that often there is a lack of information around as to whether an empirical treatment is to be started, whereas Canadian guideline recommends delaying antimicrobial therapy pending urine culture results (Baron et al., 2013; Chua et al., 2023; Kusin et al., 2023). Moreover, antibiotics chosen empirically may not be suited for treating the particular uro-pathogen found in the culture, and most of the times, cultures return negative requiring no antibiotic therapy, which is undesirable, especially in the AMR era (Kusin et al., 2023).

It is promising to notice the emergence of innovations including point-of-care tests (PoCTs) and artificial intelligence (AI)-assisted diagnostics that offer accurate and at the same time rapid results that aid in guided therapy. However, their implementation remains limited unfortunately, primarily due to barriers in clinical adoption mainly because of excessive cost, governance, and policy gaps (Korte et al., 2020; Herter et al., 2022; Van Der Pol et al., 2022; Hermans et al., 2024; Naik et al., 2024; Tolley et al., 2024).

Considering the significant advantages in the field of advanced diagnostic technologies available for rapid diagnosis of UTIs, this review critically investigates the current diagnostic landscape for UTIs, focusing on how diagnostic delays, inaccuracies, system-level barriers, and gaps exacerbate UTI-mediated AMR spread. We also argue that while diagnostic innovations are important for a positive impact on stewardship, their impact on AMR control is entirely dependent on effective implementation; in its absence, advances remain largely inconsequential. We review UTI epidemiology, management, diagnostic platforms, and their advancement through the lens of stewardship and implementation barriers. We also highlight the importance of converging innovation, practice, and policy to address the persistent challenges in UTI management.

## Published research papers selection

### Literature identification and selection

This is a critical narrative review with the aim of synthesising and contextualising evidence across urinary tract infection (UTI) epidemiology, diagnostic technologies, antimicrobial resistance (AMR), and real-world implementation barriers, and therefore, a formal systematic review or meta-analysis was not carried out.

### Strategy and data sources

Utilising PubMed and Google Scholar, literature search was performed covering publications from 1990 to 2024. Combinations of keywords and phrases including but not limited to urinary tract infection, UTI diagnosis, urine culture, point-of-care testing, molecular diagnostics, biomarkers, artificial intelligence, antimicrobial resistance, and antimicrobial stewardship were used. To enable thematic organisation and screening for relevant content, separate EndNote libraries were maintained for each major topic area that included epidemiology, diagnostics, PoCT, AMR, AI, and implementation.

In addition to peer-reviewed research papers, review papers, guideline documents, surveillance reports, and policy-relevant resources were also consulted where appropriate. Information from manufacturers’ websites, the Organisation for Economic Co-operation and Development (OECD), and reputable clinical microbiology and public health agency websites were included selectively that provided relevant content on diagnostic availability, regulatory frameworks, and implementation considerations.

### Screening of literature

A total of 230 publications were retrieved across all topic areas. Duplicate records were removed as identified across databases during EndNote curation. Following title and abstract review, 161 publications were selected for full-text evaluation and inclusion inclusion in the current critical narrative review.

### Criteria for including studies for critical narrative review

Studies that (a) addressed UTI epidemiology, diagnostic performance, clinical utility, or diagnostic innovation, (b) examined relationships between diagnostic practices, antimicrobial prescribing, or resistance, (c) provided clinically, microbiologically, or health-system–relevant insights, including implementation, policy, or stewardship considerations were included.

Studies were excluded when they were (a) not relevant to UTI diagnosis, management, or AMR, (b) editorials, commentaries, or opinion pieces lacking substantial data or critical analysis, and (c) redundant publications that did not add new conceptual, methodological, or contextual value.

### Synthesis of content for the review

Given that this is a critical narrative as well as an integrative review, a formal risk-of-bias assessment or quantitative quality scoring framework was not applied. Instead, studies were appraised qualitatively, emphasising clinically validated diagnostic studies, large observational studies, systematic reviews, guideline-based reports, national and international AMR surveillance data, and evidence addressing real-world implementation, health-economic, and policy dimensions. The manuscript was synthesised to highlight diagnostic strengths and limitations, translational gaps between innovation and clinical practice, and implications for antimicrobial stewardship and AMR mitigation.

## UTI burden and AMR

### Epidemiology

Urinary tract infections (UTIs) pose serious threats to public health in clinical and community settings being the most common outpatient clinical cases where 50%-60% of adult women experience UTI at least more than once in their lifetime owing to their anatomical predisposition (Alós, 2005; Tandogdu and Wagenlehner, 2016; Wagenlehner et al., 2016; Medina and Castillo-Pino, 2019; Mortazavi-Tabatabaei et al., 2019; Davey et al., 2020; Czajkowski et al., 2021; Paul et al., 2021; Yang et al., 2022a). In general, prevalence increases with age, with a distinct peak among women aged 14–24 years and the prevalence is highest in women over 65 years (20%), whereas the overall prevalence is 11% (Chu and Lowder, 2018; Kranz et al., 2018; Abou Heidar et al., 2019). UTI among male patients is regarded rare; however, studies on male urinary tract reported that different types of UTIs occur in men including cystitis, orchitis, prostatitis, pyelonephritis, and un-differentiated urinary tract infections (Brumfitt et al., 1991; Griebling, 2005; Farrell et al., 2021; Soudais et al., 2021). Nearly 10% of the men suffered from cystitis, and the incidence in the community is reported to be 0.9–2.4 cases per 1000 under <55 years of age and 7.7 per 1000 at and/or over 85 years of age, as reviewed by Farrell et al. (2021).

UTIs are considered a significant health concern in children as well (Falup-Pecurariu et al., 2020; Oliveira and Mak, 2020; Renko et al., 2022; Kassogue et al., 2023), and it has been reported that 6%-8% of the febrile infants and unwell older children in general practice have UTIs (Naseri et al., 2020; Cesca et al., 2022). It is encountered predominantly in female children and uncircumscribed male children and infants (Suh et al., 2021; Renko et al., 2022; Daniel et al., 2023). Factors that influence the concentration of microflora, urine stasis, and anatomical predisposition influence children getting affected by UTIs; for example, usage of nappies in infancy, shorter female urethral distance and presence of foreskin in uncircumcised men, toilet training during toddler years, and bladder stasis promote UTIs in children (Tullus, 2019; Renko et al., 2022; Daniel et al., 2023).

Studies involving infants below 8 weeks of age found a UTI prevalence ranging from 13.6% in febrile infants (Lin et al., 2000; Naseri et al., 2020; Suh et al., 2021). In addition, studies also investigated the reliability of urinalysis and revealed a prevalence of 2.9% for girls and 1.5% for boys that aged below 2 years (Bachur and Harper, 2001; Öztürk and Murt, 2020; Suh et al., 2021; Daniel et al., 2023). UK and Sweden stated that 10% of the girls and 3% of the boys would have had experienced UTI before the age of 16 (Desai et al., 2016; Williams and Craig, 2019; Öztürk and Murt, 2020; Renko et al., 2022; Daniel et al., 2023; Kassogue et al., 2023) and recommended the diagnosis of underlying congenital abnormalities and recurrent infections to prevent renal scarring and long-term renal damage (Desai et al., 2016; Williams and Craig, 2019). Uro-pathogenic *E. coli* has been reported to be attributable for 80%-90% of UTIs in children (Kaufman et al., 2019; Nguyen et al., 2022). In women, adolescent women experience UTIs due to sexual activities affecting the microflora residing in the urethral surface (Öztürk and Murt, 2020; Nguyen et al., 2022).

A plethora of studies suggest that Gram-negative bacilli belonging to *Enterobacteriaceae* are attributable for most of the UTIs as reviewed by Mortazavi-Tabatabaei et al. (2019); however, other organisms including *Staphylococcus aureus* (Lafon et al., 2019), *Enterococci*, *Enterobacter*, and *Pseudomonas aeruginosa* are considered potentially important as well. The popularly known ESKAPE (*Enterococcus*, *S. aureus*, *K. pneumoniae*, *A. baumannii*, *P. aeruginosa*, and *E. coli*) organisms are of significant concern due to nosocomial infections globally, and it is a worry when it comes to UTI as well (Zhen et al., 2019; Nguyen et al., 2022; Daniel et al., 2023).

Therapeutic guidelines from different countries recommend drugs such as nitrofurantoin, trimethoprim-sulphamethoxazole, and fosfomycin for uncomplicated UTI in women (Bell et al., 2022; Benning et al., 2023); however, resistance in Gram-negative organisms particularly in *E. coli* and *Klebsiella* species has necessitated a requirement for next-generation antibiotics (Benning et al., 2023). In Australia, UTI was the most common infection that was prophylactically treated (34.1%) with antibiotics that included asymptomatic UTI, cystitis, and catheter-associated UTI (Australian Commission on Safety and Quality in Healthcare, 2021). The top five UTI organisms or uro-pathogens causing antimicrobial resistance are reported to be *Escherichia coli*, *Klebsiella pneumoniae*, *Proteus mirabilis*, *Pseudomonas aeruginosa*, and *Enterococcus faecalis* with *E. coli* ranking the topmost resistant uro-pathogen (Mortazavi-Tabatabaei et al., 2019; Australian Commission on Safety and Quality in Healthcare, 2021; Cuningham et al., 2021; Aslan and Paterson, 2024).

Particularly, the healthcare-associated UTIs range as high as 9%-10% (Tandogdu and Wagenlehner, 2016; Wagenlehner et al., 2016). A data analytical study was conducted to investigate the global burden of UTIs to depict the incidence, mortality, and disability-adjusted life years (DALYs) encompassing 204 countries and territories from 1990 to 2019 based on socio-demographic status, nation’s region, sex, and age (Yang et al., 2022a). This study revealed important observations about UTIs and the associated sequel worldwide where there had been 404.61 million cases, 236,790 deaths, and 520,200 DALYs estimated globally in 2019. The death rates have been recorded from 1990 to 2019, and they observed a 2.4 times growth in deaths with an increasing age-standardised mortality rate (ASMR) from 2.77/100,000 to 3.13/100,000, and the age-standardised incidence rate (ASIR) was 3.6 times higher in women than men. The burden rate was observed to increase with age, and the study also emphasised that among 21 Global Burden of Disease study regions, Andean Latin America, Tropical Latin America, and Australasia experienced the highest ASIR in 2019, which ranged from 96.63 to 131.64 per 1,000 person-years (Yang et al., 2022a). Studies from Australia have reported healthcare-associated UTIs as high as 25,200-68,735 infections annually with deaths between 259 and 1772 per annum, DALYs 259-1772, years of life lost (YLL) 3899-26,704 and years lived with disability (YLD) 1745-11,659 (Lydeamore et al., 2022). It is worth noting that UTIs consistently top the list as the primary reason for antibiotic administration in Australian Residential Aged Care Facilities (RACFs), according to yearly national point prevalence surveys (Lydeamore et al., 2022).

UTI being a global burden is a known fact. Despite this awareness, UTI continues to be a significant contributor of a large share of disproportionate antibiotic prescriptions at both community and hospital levels, which are often provided without systematic pathogen confirmation (Australian Commission on Safety and Quality in Healthcare, 2021; Rowe et al., 2022). For instance, studies from aged care and emergency settings have reported that 60% of UTIs are diagnosed symptomatically and treated empirically, with diagnostic uncertainty driving inappropriate antimicrobial use (Rowe et al., 2022; Bono et al., 2023). It was also highlighted by van der Pol et al. (van der Pol et al., 2022) that prescriptions without proper diagnosis and/or symptomatic treatment in general practice accelerates AMR in those settings. Furthermore, review studies (Cuningham et al., 2021; Paul et al., 2021; Tolley et al., 2024) have highlighted the emergence and existence of AMR uro-pathogens from different countries. These studies reveal a persistent gap between UTI burden, diagnostic inaccuracies, and diagnostic implementation failures. This is concerning particularly in the presence of WHO high-priority pathogens such as *E. coli* and *Klebsiella* (CDC, 2024a; WHO, 2024). It should also be noted that these are also prioritised by CDC and other national AMR research agencies like the Australian Commission on Safety and Quality in Healthcare (Healthcare, 2023; CDC, 2024a). Furthermore, diagnostic inadequacy and inaccuracies contribute to the rise of resistant uro-pathogens that compromise the first-line therapies that are used in UTI (Chua et al., 2023; Kusin et al., 2023). All these epidemiological studies underscore the pressing need for accurate, accessible, and timely diagnostic tools. Without addressing diagnostic uncertainty, UTI-related antibiotic overuse will continue to drive antimicrobial resistance across healthcare settings.

These epidemiological patterns collectively underscore that (1) UTI burden is highly heterogeneous across populations and healthcare settings, (2) the symptoms are often non-specific, and (3) diagnostic confirmation is inconsistently pursued. This heterogeneity directly contributes not only to diagnostic uncertainty but also to the clinical reliance on empirical antimicrobial therapy, particularly in primary care, aged care, and paediatric settings. Consequently, diagnostic tools and methods with broad applicability and adaptability are necessary, whereas UTI burden cannot be meaningfully addressed without parallel improvements in diagnostic strategies that support antimicrobial stewardship preventing unnecessary antibiotic exposure.

## Clinical management and antimicrobial resistance

The clinical management of UTIs remains significantly reliant on empirical antibiotic therapy as stated earlier, particularly in community and aged care settings. In adults and elderly, UTIs are classified suspected, proven, recurrent, and/or relapsed (Lim and Bennett, 2022). In general, residents in residential aged care facilities (RACFs) predominantly present with localised urinary tract signs and symptoms, although some may develop acute illness with non-specific systemic manifestations, including behavioural changes, functional decline, falls, and malaise, as reviewed by Lim and Bennett (2022). It is often reported that the behavioural changes may be the only observable symptom. However, a review on male UTI management by Soudais et al. (2021) stated that the frequency, diagnosis, classification, and treatment of male UTIs remain unclear across countries, which still requires more detailed studies, and it is reported that the male UTIs are classified based on the intensity and the tolerance of patients’ symptoms (Caron et al., 2018).

Guidelines such as those from the IDSA, NICE, and national frameworks in Australia and Canada recommend first-line antimicrobials including nitrofurantoin, fosfomycin, and trimethoprim-sulfamethoxazole based on historic pathogen susceptibility (Davey et al., 2020; Chua et al., 2023; Kusin et al., 2023) that are bypassed in practice by General Practitioners (GPs) to contend with mainly diagnostic delays and non-specific symptoms and of course uncertainty about resistance patterns.

Multiple studies confirm fluoroquinolones and broad-spectrum beta-lactams continue to be overused even for uncomplicated UTIs due to perceived efficacy and easy management. An Australian study reported that antibiotics were prescribed in 86% of GP index consultations for UTIs. This prescribing rate was primarily driven by adults, who were more likely to receive antibiotics at the index consultation compared with children under 16 years (Davey et al., 2020). It should be noted that broad-spectrum antibiotics including cephalosporins, beta-lactam penicillins, and quinolones are frequently prescribed, not just for UTI treatment but also for prophylaxis and even for asymptomatic bacteriuria (ASB), a scenario where antibiotics are unnecessary (Crnich et al., 2015; Australian Commission on Safety and Quality in Healthcare, 2021; Rowe et al., 2022). For example, cephalexin is prescribed for those over the age of 95 years with repeats at a rate of 38.2%; amoxycillin-clavulanate (age 80-84) at 51.9%, and ciprofloxacin (age 90-94) at 28.6% (Australian Commission on Safety and Quality in Healthcare, 2021). Furthermore, family pressure to prescribe broad spectrum antibiotics is reported to be a reason as well (Lim and Bennett, 2022).

**Figure 1:**
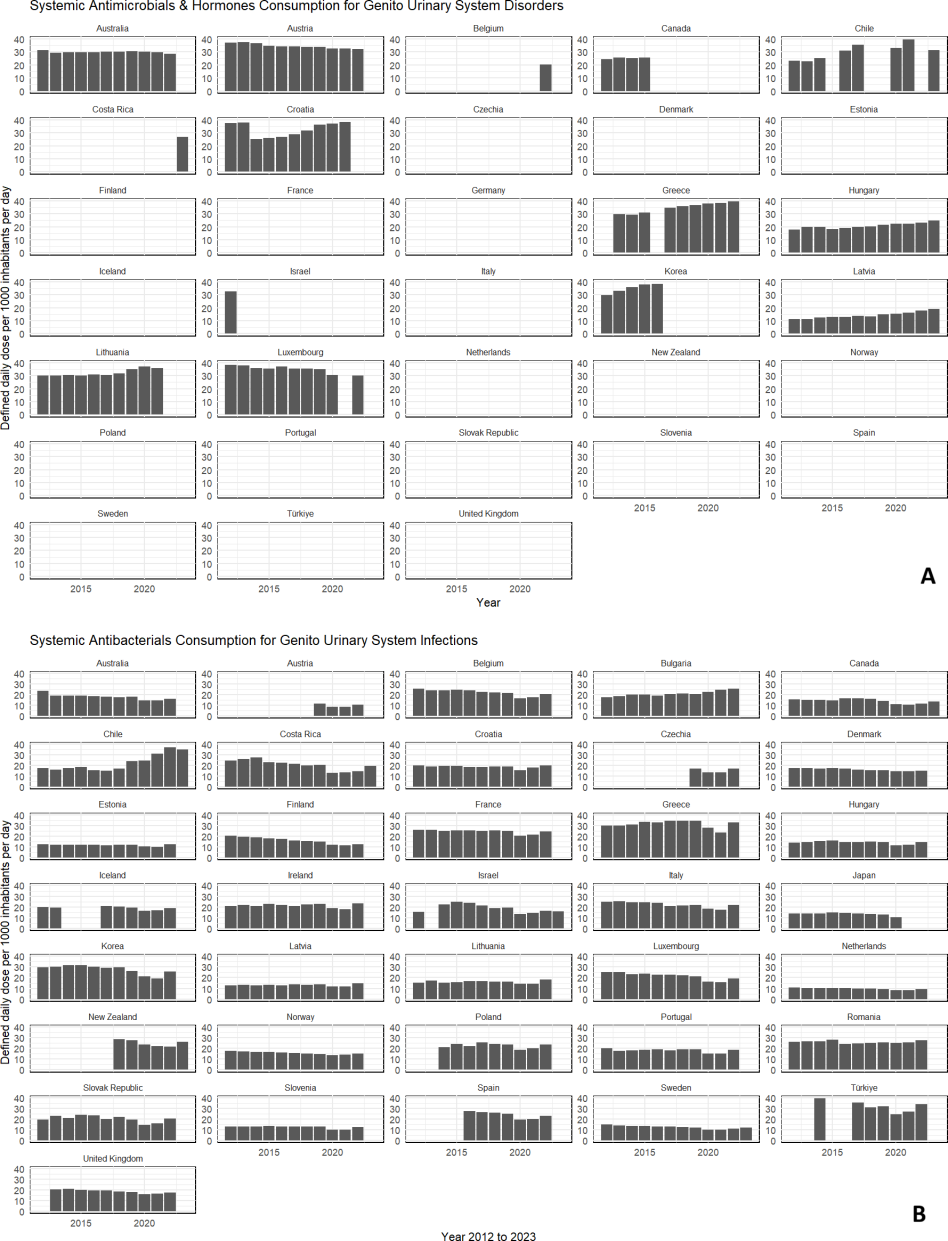
**(A)** Total volume of systemic antimicrobial and hormone use recorded worldwide by the Organisation for Economic Co-operation and Development (OECD) database for genito-urinary system disorders. Limitations: The data and information were downloaded and recorded for the years 2012 to 2023; however, some countries did not have data available for several of those years in the OECD database. **(B)** Total volume of systemic antibacterial use recorded worldwide by the Organisation for Economic Co-operation and Development (OECD) database for genito-urinary infections. Limitations: The data and information were downloaded and recorded for the years 2012 to 2023; however, some countries did not have information for several years, and data were not available for certain countries in the OECD database. .

Yet another noteworthy scenario is the differences in the AMR rates and the demographics in different parts of the world. For example, Antimicrobial Resistance and Use in Australia (AURA) reported differences in the resistance rates among different organisms in Australia (Australian Commission on Safety and Quality in Healthcare, 2021). Resistance in Gram-negative pathogens is reported to continuously increase towards common antimicrobials. For example, *Escherichia coli*, one of the most common pathogens causing UTI, has been reported to have become resistant to common agents used for treating *E.coli*-UTI and septicaemia in the community in otherwise healthy people (Australian Commission on Safety and Quality in Healthcare, 2021). The report has flagged differences in AMR rates between hospital settings and RACFs in Australia. For instance, *E. coli* resistance towards first-generation cephalosporins is 35% in RACFs compared with 22% in hospitals. Similarly, 26% of resistant *Staphylococcus aureus* is encountered in RACFs as opposed to 22% in hospitals. Likewise, extended beta-lactamase *E. coli* infection is a threat, which often is encountered in the aged population that becomes untreatable as a consequence of prophylaxis and unnecessary antibiotic prescriptions (Australian Commission on Safety and Quality in Healthcare, 2021; Cuningham et al., 2021). In addition, antibiotic prescription rates within the community are twice as high as in some of the countries affiliated with the Organisation for Economic Co-operation and Development (OECD) (Lydeamore et al., 2022). **Figures 1a, b** demonstrate the volume of antibiotics consumed by different countries reported by the OECD database where **Figure 1a** represents the total antimicrobial and homone consumption for genito-urinary system disorders and **Figure 1b** represents the antibacterial consumption for genitourinary system infections. Data had been collected and collated from 2001 to 2023 where information was not available for some countries as well as years for comparison.

## Diagnosis innovations for UTI: practice, performance, and pitfalls

### Practice

A complete urinalysis generally involves a set of diagnostic procedures including microscopy (for the presence of cellular segments and sediments as well as microbes), biochemical analysis, staining, quantitative microbiological analysis, and culture and antimicrobial susceptibility tests following the recovery of the particular pathogen from the suspected specimen for UTI (Roxe, 1990; Brubaker et al., 2023; Zamudio et al., 2024). Any one individual procedure is always considered inadequate for an effective diagnosis, and usually it is a combination of two or more test procedures used to diagnose UTI (Wilson and Gaido, 2004; Brubaker et al., 2023). Collection and storage of urine specimens play an important role in providing a report that is of good diagnostic value. A clean catch of mid-stream urine is a recommended procedure for UTI diagnosis, and refrigerating the specimens soon after collection is always recommended. Delay more than 2 h is usually regarded as not suitable for analysis (Wilson and Gaido, 2004; Simerville et al., 2005; Brubaker et al., 2023). Physical properties include colour and odour of the urine specimen, which are often influenced by medicines, food, infection, and other associated health conditions (Chu and Lowder, 2018; Brubaker et al., 2023). Techniques and methods used for diagnosing UTI have evolved over the years with the invention of new technological platforms and tools for physical, chemical, and microbiological analysis.

The main types of diagnostic platforms include the following:

1. Conventional point-of-care dipstick analysis
2. Culture-based methods for identifying the pathogen species causing UTI
3. Molecular methods that utilise nucleic acid amplification such as polymerase chain reaction (PCR), real-time PCR, isothermal amplification (carried out on standard benchtop equipment, or on biosensors/lab-on-a-chip devices), and host-specific biomarkers.

Using dipsticks as a tool for diagnosing UTI has been viewed as a rapid and less labour-intensive procedure, and often this is served as a screening tool for UTI (Dadzie et al., 2019). Several commercial manufacturers produce urine dipstick assays for the preliminary diagnosis of UTIs. These dipsticks comprise multiple biochemical tests, including detection of red blood cells (RBCs), bilirubin, urobilinogen, glucose, ketones, protein, pH, nitrites, leukocyte esterase, and specific gravity. Among the 10 parameters assessed, leukocyte esterase and nitrite are the primary markers used to infer the presence of a UTI, as they reflect host inflammatory response and bacterial nitrate reduction, respectively. However, reliance on these two indicators alone has inherent limitations, as their sensitivity and specificity vary with pathogen type, urine concentration, and patient factors, potentially leading to false-negative or false-positive results. The order of analytes on the dipstick may vary depending on the manufacturer. However, it should be acknowledged that other conditions also may be attributable for the presence of leucocytes in the urine and not all urinary bacterial infections produce nitrites in urine (Dadzie et al., 2019). Nevertheless, there is a positive correlation between the presence of nitrites and UTI. Furthermore, dipstick results can be impacted by byproducts that are excreted as part of specific foods, medication, and other underlying medical conditions that may lead to faulty interpretation of the results (Simerville et al., 2005; Lehmann et al., 2011; Mambatta et al., 2015; Bacârea et al., 2021; Zhao et al., 2024).

Culture-based methods are an essential part of UTI diagnosis as this is considered the gold standard for diagnosing UTI and usually the results are reported after 48-72 h with antimicrobial susceptibilities (Dadzie et al., 2019). At times of repeated UTIs with negative urine culture, enrichment of urine to detect the uro-pathogen has been reported to be very useful (Cao et al., 2022). Often, the routine culture methods involve inoculating sterile enriched bacterial agar media (non-selective) such as blood agar media and selective media such as MacConkey agar media with urine specimen for differential growth characteristics (Graham and Galloway, 2001; Karah et al., 2020). In the place of MacConkey, cysteine lactose electrolyte deficient (CLED) media has been used as a standard media for urine cultures of late (Karah et al., 2020). In the light of microscopic evidence of fungal elements and/or yeast buds visualised in microscopy, in addition to routine bacterial media, Sabouraud agar media should be added to rule in yeast infection (Karah et al., 2020). Interpretation of urine culture results depends on both the number of colonies and the bacterial morphologies observed. When a culture plate shows more than 100 colonies of a single bacterial type, this corresponds to a bacterial load of ≥10^5^ colony-forming units (CFU)/mL, which is considered indicative of a urinary tract infection. In such cases, the isolate is further identified to the species level, and antimicrobial susceptibility testing is subsequently performed (CDC, 2024b). A colony count of less than 10 is usually considered low probability of an UTI. Also, if there is more than two types of colonies observed on the culture plate, a repeat sample is requested (CDC, 2024b). On the other hand, if there are two types of colonies found at a colony forming unit (CFU) of 10^5/^mL, both types are identified and subjected to antimicrobial susceptibility tests (Karah et al., 2020). Other clinical diagnosis of UTI also involves enzymatic assays such as catalase testing using urine samples where the limitation of the test is the low specificity (Khekade et al., 2022) and pathogen specific assays, which means that urine culture is necessary for conducting this test (White et al., 2021).

**Table 1** was adapted from https://www.community.healthcare.mic.nihr.ac.uk/reports-and-resources/horizon-scanning-reports/point-of-care-testing-for-urinary-tract-infections with some modifications and provides a comprehensive list of PoC tests utilised for UTI screening, and several research studies have evaluated these PoC tests in real time. **Table 1** lists the dipstick brands, automated, semiautomated, and enzyme assays that are currently available in the market and evaluated by researchers (Rosenberg et al., 1992; Waisman et al., 1999, Waisman et al., 1999; Scarparo et al., 2002; Arienzo, 2016; Arienzo et al., 2016, Arienzo et al., 2020; Oviaño et al., 2021). Some companies manufacture semiautomated dipstick assays where the assay results can be read using a colorimetry reader and the data can be transferred to the laboratory management system.

**Table 1:** This table lists the commercially available point-of-care tests for urinary tract infection with company names, turnaround times, description of the device, sample volume, storage conditions, method or principle on which the device functions, portability, and additional equipment requirement for running the devices adapted from https://www.community.healthcare.mic.nihr.ac.uk/reports-and-resources/horizon-scanning-reports/point-of-care-testing-for-urinary-tract-infections with slight modifications.

Product	Reference /research studies	Physical appearance	Manufacturer / location	Analysis time	Description of device	Type of sample analysed (volume of sample)	Portable	Additional equipment required	Positive result outcomes	Storage temp. (celsius)	Method principle
CULTURE BASED DEVICES											
FLEXICULT ™	Documentation - SSI Diagnostica	#VALUE!	Statens Serum Institut Diagnostica / Denmark	24 hours	Chromogenic agar plate with 6 segments - 5 evaluating antibiotic sensitivities and 1 control segment	Midstream urine sample ; volume not specified	Portable	Incubator	1. Bacterial growth semi-quantification 2. Species identification 3. Antibiotic Susceptibility	2-8 °C	Culture and susceptibility testing
Uricult Trio	Aidian Academy	#VALUE!	Orion Diagnostics / Finland	16-24 hours when incubated at 36.8°C or 1-3 days at room temperature	Plastic slide with CLED/MacConkey + E.coli agar medium	Randomly voided, midstream, clean-catch and catheterized samples ; sample volume not specified	Portable	Incubator	1. Bacterial growth semi-quantification 2. Species identification	15-25°C	Culture
DipStreak (Chromostreak)	Scarparo et al., 2002	#VALUE!	Novamed / Israel	18-24 hours	Plastic paddle with two opposing agar media (UriSelect3 chromogenic agar and MacConkey), housed in a closed transparent plastic tube	Midstream urine sample ; No sample volume	Portable	Incubator	1. Bacterial growth semi-quantification 2. Species identification	2-8 °C	Culture
DiaSlide	Rosenberg et al., 1992	#VALUE!	Novamed / Israel	24 hours	CLED/MacConkey or UriSelect3 chromogenic agar and MacConkey - Petriplates hinged opposing each other.	Midstream urine sample ; No sample volume	Portable	Incubator	1. Bacterial growth semi-quantification	15-25°C	Culture
onSite	ThermoFisher Scientific		Trek Diagnostics System / USA	Not specified	MacConkey agar + one of TSA/Columbia CNA/CLED agar hinged with two petri plates	Midstream urine sample ;	Portable	Incubator	1. Bacterial growth semi-quantification 2. Species identification	2-25°C	Culture
Micro Biological Survey (MBS) POCT	Arienzo et al., 2016	#VALUE!	MBS Diagnostics Ltd. / UK	5 hours	Portable computer powered +vials and redox indicator	Midstream Urine (1mL)	Portable	incubator +reader	Semi quantitaive	4-25°C	Calorimetric - colour change
ENZYMATIC ASSAY											
UriScreen	Pezzlo et al., 1992	No speific image	Savyon Diagnostics Ltd / Israel	2 minutes	Enzymatic (catalase) test	Midstream urine sample ; (1.5 -2 mL)	Portable	Portable	Detects bacteriuria / pyuria	Room temp (10-28°C)	Tube Assay
Accutest® Uriscreen™	Waisman, et al., 1999		Jant Pharmacal Corp / USA	2 minutes	Enzymatic (catalase) test	Midstream urine sample ; (1.5 -2 mL)	Portable	Portable	Detects bacteriuria / pyuria	Room temp (10-28°C)	Tube Assay
Uranotest® Uriscreen™	https://www.uranodiagnostics.com/en/products/uranotest-uriscreen		Urano Diagnostics / Spain	2 minutes	Enzymatic (catalase) test	Midstream urine sample ; (1.5 -2 mL)	Portable	Portable	Detects bacteriuria / pyuria	Room temp (10-28°C)	Tube Assay
AUTOMATED/SEMI AUTOMATED URINE ANALYSERS											
Product			Manufacturer / Location	Analysis Time	Description of device	Voume of sample	Portable	Print out results	Additional equipment required	Positive result outcomes*	Method Principle
URI TEX	https://cormaydiagnostics.com/products/urinalysis/analyzers-7/uri-tex	#VALUE!	Cormay / Poland	60 seconds	Semi-automated portable analyser	Dipstick -Not applicable	Portable	Optional software	Urine dipstick strips	10 parameters	Reflectance photometer
Uro-Dipcheck ® 240e	https://healthmanagement.org/products/view/automated-urine-analyzer-portable-uro-dipcheck-240e-erba-diagnostics-mannheim	#VALUE!	Erba Diagnostics Mannheim / Germany	55 seconds	Automated portable analyser	Dipstick -Not applicable	Portable	Print	Urodip 10 e, Dekaphan test strips	10 parameters	Reflectance photometer
Convergys® Urostar 100	https://empiricahealthcare.com/en/product/semi-automated-urine-analyzer-convergys-urostar-100/	#VALUE!	Invergent Technologies / UK	1-2 minutes	Automated portable analyser	Dipstick -Not applicable	Portable	Print	Convergys® Urostar Strips or CM	10 + Creatinine & Albumin and Microalbumin to Creatinine ratio	Semi-quantitative reflex photometer with 3 x LED + color detector
AUTION ELEVEN™ AE-4020	https://www.arkray.eu/english/upload/docs/ae-4020.pdf	#VALUE!	Arkray / Japan	7 seconds	Semi-automated portable analyser	Dipstick -Not applicable	Portable	Print	AUTION test strips	10 + automatic protein/creatinine and albumin/creatinine calculation	Reflectance photometer
Aution Micro	https://healthmanagement.org/products/view/portable-urine-analyzer-aution-micro-menarini-diagnostics-1	#VALUE!	A. Menarini Diagnostics / Italy	45 seconds	Automated portable analyser	Dipstick -Not applicable	Portable	Print	AUTION Sticks 10EA, Uriflet 9UB	10 parameters	Wavelength Reflectance Method -Single and double
URIT-30	https://healthmanagement.org/products/view/all/portable-urine-analyzer-urit-30-urit-medical-electronic-group	#VALUE!	URIT / China	60 seconds	Automated portable analyser	Dipstick -Not applicable	Portable	Print	URIT 11G/10G urine reagent strips	10 parameters	Dual Wavelength Reflectance Method
BioDoctor BS-502	https://healthmanagement.org/products/view/portable-urine-analyzer-biodoctor-bs-502-infopia	#VALUE!	Bionics Co. Ltd / Korea	5 seconds	Semi-automated portable analyser	Dipstick -Not applicable	Portable	Not specified	Urine dipstick strips (unspecified brand) + iOS device - Results sent to smart phone device	10 + free radicals	Details not specified
AS120	https://www.medicalexpo.com/prod/chungdo-pharm-co-ltd/product-68041-438014.html	#VALUE!	ChungDo Pharm. Co., Ltd / China	Details not specified	Automated portable analyser	Dipstick -Not applicable	Portable	Not specified	Self-Stick + test strips	10 + ascorbic acid	Details not specified
E-Reader 120	https://healthmanagement.org/products/view/all/semi-automatic-urine-analyzer-e-reader-120-accubiotech	#VALUE!	AccuBioTech. Co., Ltd / China	Details not specified	Semi-automated portable analyser	Dipstick -Not applicable	Portable	Not specified	ABT-UM-A33/34 test strips	10 + ascorbic acid	Reflectance photometer
BC401	ThermoFisher SCIENTIFIC	#VALUE!	Contec Medical Sysems™	Details not specified	Automated portable analyser	Dipstick -Not applicable	Portable	Not specified	Details not specified	10 + ascorbic acid	Details not specified
Uryxxon Relax	https://www.mn-net.com/urine-test-strip-analyzer-uryxxon-relax-93088	#VALUE!	Macherey Nagel / Germany	30 - 60 seconds	Automated analyser	Dipstick -Not applicable	Portable	Not specified	Medi-Test Uryxxon Stick 10	10 parameters	Reflectance photometer
Urisys 1100	https://diagnostics.roche.com/global/en/products/instruments/urisys-1100-analyzer-ins-812.html	#VALUE!	Roche Diagnostics Ltd / Switzerland	70 seconds	Semi-quantitative analyzer	Dipstick -Not applicable	Portable	Print	CHEMSTRIP test strips	10 parameters	Reflectance photometer
Clinitek Status+	https://www.siemens-healthineers.com/urinalysis-products/urinalysis-systems/clinitek-status-analyzer	#VALUE!	Siemens Healthcare /Germany	60 seconds	Automated portable analyser	Dipstick -Not applicable	Portable	Print	CLINITEK Multistix® urine test	10 + protein/creatinine and albumin/creatinine calculation	Reflectance photometer
Urilyzer ® 100 Pro	https://www.analyticon-diagnostics.com/en/products_and_solutions/urine_diagnostic/analyzer/urilyzer_100.html	#VALUE!	Analyticon Biotechnologies AG / Germany	60 seconds	Automated portable analyser	Dipstick -Not applicable	Portable	Print	CombiScreen® 5/7/11SYS PLUS	10 parameters	Reflectance photometer
BIOSYNEX UTI Self-Test	BIOSYNEX Urinary Infection Self-Test	No speific image	BIOSYNEX / France	60-120 seconds	Automated portable analyser /Manual	Dipstick -Not applicable	Portable	Print	Not spcified	4 parameters leukocytes, nitrites, blood, proteins	Manual Visualisation
Immunoassays/Lateral flow platforms											
Rapidbac	Stapleton et al., 2015		Silver Lake Research / USA	15-30 minutes	Manual	0.5 mL	Portable	No	lateral flow kit	Bacteriuria	Visual

10 paramters include : - Specific Gravity, pH, Leukocytes, Nitrite, Protein, Glucose, Ketone, Urobilinogen, Bilirubin, Erythrocytes.

Point-of-care testing for urinary tract infections — NIHR Community Healthcare MIC Website accessed 03/07/2025

### Practice and pitfalls

In contrast to conventional culture-based diagnostics, which prioritise organism recovery and susceptibility determination but are limited by prolonged turnaround times, advanced molecular and proteomic platforms aim to accelerate pathogen identification and resistance inference. However, their clinical performance, evidence base, and real-world utility vary considerably depending on platform design, clinical context, and implementation setting.

While urine dipsticks can be a rapid diagnostic for UTI, a number of studies have explained why urine dipstick alone cannot determine the pathophysiology of UTI (St John et al., 2006; Mambatta et al., 2015; Dadzie et al., 2019; Walker et al., 2019; Bacârea et al., 2021). In contrast, studies reported that dipstick can be a rapid test ruling in UTI (Whiting et al., 2005; Bellazreg et al., 2019; Suresh et al., 2021), whereas some studies reported dipstick to be moderately sensitive and useful. It should be noted that a clean midstream urine is necessary for accurate diagnosis and results will vary depending on the void (Pernille et al., 2019).

Although tools for rapid screening are widely being used, clinical microbiology laboratories have become more reliant on molecular diagnostic tests and nucleic acid amplification tests (NAAT) including (endpoint) PCR, real-time PCR (rtPCR), multiplex PCR, and isothermal amplification technologies, such as loop-mediated isothermal amplification (LAMP) (Notomi et al., 2000; Nagamine et al., 2002; Tanner et al., 2012), recombinase polymerase amplification (RPA) (Piepenburg et al., 2006; Escadafal et al., 2013), helicase-dependent amplification (HDA) (Vincent et al., 2004; Jeong et al., 2009), and strand invasion-based amplification (SIBA) (Eboigbodin and Hoser, 2016). Although isothermal amplification technologies are typically not quantitative (as real-time PCR can be under conditions), they are typically quite faster, as they can detect the presence of a pathogen within a few minutes. For UTIs, these platforms have been proven to remarkably improve the accuracy of UTI diagnosis (Robledo et al., 2022), although they are time consuming compared with dipstick assays. These tests are regarded as accurate as standard urine culture tests, and further refinement of them has been regarded as an advantage to prevent the emergence of AMR (Robledo et al., 2022). Multiplex and rtPCR have been applied to diagnose uro-pathogens directly from urine in symptomatic patients and for detecting polymicrobial UTIs with superior sensitivity and specificity (Lehmann et al., 2010; Wojno et al., 2020). Qualitative PCR has been run using SeptiFast^®^ Real-Time PCR where the turnaround time for results were reported to be much faster (Lehmann et al., 2011; Zhao et al., 2024). RtPCR had also been utilised to diagnose UTI as well as antimicrobial resistant UTI using commercially available kits among cancer patients, which was reported to be useful for guided therapy and rapid detection (Cybulski et al., 2013; Quarton et al., 2024; Tomiyama et al., 2024; Zamudio et al., 2024). Few studies have used quantitative as well as semiquantitative PCRs in routine hospital settings (Kapoor et al., 2024; Khanal et al., 2024; Nikiema et al., 2024). Similarly, matrix-assisted laser desorption ionisation time-of-flight (MALDI-TOF) assay has been used to detect uro-pathogens (Sánchez-Juanes et al., 2018; Frickmann et al., 2024; Muthusamy et al., 2024; Sánchez et al., 2024). Biosensors have revolutionised the pathogen detection that includes uro-pathogens, as reviewed recently (Sharma et al., 2021). **Table 2** details the biosensors used for uro-pathogens and NAATs available for uro-pathogens that were evaluated by researchers.

**Table 2:** Nucleic acid assisted tests (NAAT) for urinary pathogen detection, immunoassays, biosensors and aptamers for urinary pathogen or uropathogen detection are listed with references.

Platform	Detection	Clinical utility (GP vs ED / AMS relevance)	Sensitivity / Specificity (real-world evidence)	References
Multiplex PCR	Multiple pathogens	High utility in ED for rapid escalation/de-escalation; limited GP uptake due to cost and workflow	Sensitivity: High (>90%); Specificity: Moderate–High (colonisation detection risk)	Akhlaghpour et al., 2024; Aljindan et al., 2024; Gleicher et al., 2024; Haley et al., 2024; Munshi et al., 2024; Samani et al., 2024; Williamson et al., 2024; Zhao et al., 2024
Titanium based MALDI bacterial chips	*Multiple organisms*	Hospital and ED laboratory adjunct post-culture; not frontline PoCT	Sensitivity: High post-culture; Specificity: High (>90/90%)	Gopal et al., 2013
Solution based circuits	*E. coli, S. aureus, P. aeruginosa*	Investigational or niche application; limited routine clinical deployment	Sensitivity/Specificity: Variable or insufficient real-world data	Ganguly et al., 2021; Yang, et al., 2022b; Lam et al., 2013
Real-Time PCR UTI	Multiple pathogens	High utility in ED for rapid escalation/de-escalation; limited GP uptake due to cost and workflow	Sensitivity: High (>90%); Specificity: Moderate–High (colonisation detection risk)	Nikiema et al., 2024; Van Der Zee et al., 2016; Lehmann et al., 2011; Lehmann et al., 2010
rtPCR Septifast RtPCR UTI	Multiple pathogens	High utility in ED for rapid escalation/de-escalation; limited GP uptake due to cost and workflow	Sensitivity: High (>90%); Specificity: Moderate–High (colonisation detection risk)	Lehmann et al., 2011
Urinary lactoferrin based electrochemical biosensor	Multiple pathogens	Promising GP/ED screening PoCT; limited real-world adoption	Sensitivity: Moderate–High; Specificity: Variable	Pan et al., 2010
Maldi-ToF- UTI	Multiple pathogens	Hospital and ED laboratory adjunct post-culture; not frontline PoCT	Sensitivity: High post-culture; Specificity: High (>90/90%)	Sánchez-Juanes et al., 2018
Aptamer based impedance biosensor	*E. coli*	Promising GP/ED screening PoCT; limited real-world adoption	Sensitivity: Moderate–High; Specificity: Variable	Chen et al., 2022; Li et al., 2018
Immunoassay	Multiple pathogens	Adjunct screening in GP/ED; not pathogen-specific	Sensitivity: Moderate; Specificity: Low–Moderate (<90%)	Stapleton et al., 2015
Multiplex PCR	Multiple pathogens	High utility in ED for rapid escalation/de-escalation; limited GP uptake due to cost and workflow	Sensitivity: High (>90%); Specificity: Moderate–High (colonisation detection risk)	Wojno et al., 2020
Biosensor	*E. coli, Proteus mirabilis, P. aeruginosa, Enterococcus spp.*	Promising GP/ED screening PoCT; limited real-world adoption	Sensitivity: Moderate–High; Specificity: Variable	Ganguly et al., 2021; Yang, et al., 2022b; Vasudevan et al., 2020; Li et al., 2018
Aptamers	*E. coli, Acinetobacter baumanii, Pseudomonas, Enterococcus, multiple organisms, drug biomarkers (ampicillin)*	Early research-stage platform; not yet routine GP or ED use	Sensitivity: High (experimental); Specificity: High (limited cohorts)	Ganguly et al., 2021; Yang, et al., 2022b; Vasudevan et al., 2020; Li et al., 2018
Aptasensor for lactoferrin	Lactoferrin biomarker	Investigational or niche application; limited routine clinical deployment	Sensitivity/Specificity: Variable or insufficient real-world data	Naseri et al., 2021
Aptamer based proteomic study	Urine biomolecules	Early research-stage platform; not yet routine GP or ED use	Sensitivity: High (experimental); Specificity: High (limited cohorts)	Dong et al., 2020
Biosensor	*E. coli*	Promising GP/ED screening PoCT; limited real-world adoption	Sensitivity: Moderate–High; Specificity: Variable	Li et al., 2018
Biosensor	*E. coli*	Promising GP/ED screening PoCT; limited real-world adoption	Sensitivity: Moderate–High; Specificity: Variable	Adrover-Jaume et al., 2020
Surface plasmon resonance	*E. coli*	Investigational or niche application; limited routine clinical deployment	Sensitivity/Specificity: Variable or insufficient real-world data	Özgür et al., 2020
Volumetric bar chart CHIP	*E. coli &Staph. aureus*	Investigational or niche application; limited routine clinical deployment	Sensitivity/Specificity: Variable or insufficient real-world data	Huang et al., 2019
Loop mediated isothermal amplification coupled biosensor	*Entero. faecalis & S. aureus*	Promising GP/ED screening PoCT; limited real-world adoption	Sensitivity: Moderate–High; Specificity: Variable	Wang et al., 2017
Photoluminiscence biosensor	*Pseudomonas aeruginosa*	Promising GP/ED screening PoCT; limited real-world adoption	Sensitivity: Moderate–High; Specificity: Variable	Vasudevan et al., 2020
Antibody independent lateral flow	*E. coli*	Investigational or niche application; limited routine clinical deployment	Sensitivity/Specificity: Variable or insufficient real-world data	Wu et al., 2023

While MALDI-TOF and NAAT-based panels demonstrate high analytical sensitivity and rapid pathogen identification under laboratory conditions, their impact on routine UTI management remains uneven. MALDI-TOF typically requires prior culture enrichment, limiting its utility as a true rapid diagnostic, whereas multiplex NAAT panels may detect colonisation or residual DNA that does not always correlate with symptomatic infection. Similarly, biosensor and omics-based host-response diagnostics show promise in early studies, but the current evidence base is dominated by small cohorts, heterogeneous study designs, and limited head-to-head comparisons with standard culture. As a result, robust data demonstrating consistent improvements in prescribing behaviour or antimicrobial stewardship outcomes remain limited.

Diagnosis of infections in infants in general is quite challenging, particularly with UTIs that are associated with bacteraemia. However, with the advent of advanced molecular biology techniques and tools, omics-based diagnostic approaches have been made possible as a breakthrough alternative to the traditional pathogen detection methods (Buonsenso et al., 2022). Host transcriptomics for differentiating bacterial and viral infections has shown to be promising in infants with high sensitivity and specificity, and it is setting the trend for rapid diagnosis of infection including UTI in infants and children (Herberg et al., 2016; Mahajan et al., 2016; Kaforou et al., 2017; Pennisi et al., 2021; Buonsenso et al., 2022; Dungu et al., 2024). As argued earlier, despite the approach’s efficiency, it is yet to be regarded as a routine diagnostic procedure due to implementation challenges.

Importantly, many emerging platforms demonstrate strong analytical performance but lack validation across diverse healthcare settings (especially remote, rural areas), healthcare systems, and patient populations, especially underrepresented population groups like elderly, children, women (especially pregnant women). This gap between technical capability and clinical deployment continues to constrain their adoption and limit their current contribution to antimicrobial stewardship.

## Point-of-care tests for UTI

The term point-of-care testing (PoCT) refers to “pathology testing performed in close proximity to a patient by a healthcare worker, usually outside the precincts of a traditional laboratory. Testing is undertaken at the time of, and for use during, a consultation or episode of care” (NPAAC, 2023). The PoCT technology or the platform in itself is distinguished from the pathology laboratory tests where the collection of specimens from patients is carried out by dedicated specimen collection centres, and the specimens get transported to a central purpose-built laboratory for requested analysis (NPAAC, 2023). The human pathology laboratory is and/or was mandatory owing to the complexity of the test procedures, the required dedicated trained medical laboratory staff that carry out the tests, bulk equipment, and the space required to install and maintain that equipment (Luppa et al., 2011; Davenport et al., 2017; Semret et al., 2018; Tolley et al., 2024). As technology advances, some of the tests that carry significant medical value such as (i) the immediate requirement of results to make medical decisions and patient monitoring and (ii) the tests that can be performed without extensive training have been devised and implemented at the patient’s bedside and at the general practice as reviewed by several studies (van der Pol et al., 2022; Sánchez-Juanes et al., 2018; Hermans et al., 2024; Tolley et al., 2024). These devices help expedite tangible improvements in medical decision making and clinical care to benefit better patient care and healthcare. In addition, point-of-care interventions (POCIs) are considered one of the effective strategies of antimicrobial stewardship (AMS). POCIs have been documented to improve patient management and patient outcomes and to provide opportunities to educate on optimal decision making by clinicians (ACSQHC, 2018). One of the POCIs is directing therapy based on the results from PoCT including rapid antigen testing, and for a small number of tests, it has been proven to be effective to improve the choice of empirical therapy (ACSQHC, 2018; Navarro et al., 2020).

## Rapid diagnostics—PoCTs, molecular platforms, and biomarkers—are these implementable?

The PoCTs and the NAATs are frequently promoted as transformative tools for UTI diagnosis owing to their rapid turnaround times, portability, and ability to support early clinical decision-making. Advanced technologies such as biosensors, multiplex PCR assays, optical detection platforms, and host-response-based tests have demonstrated robust performance under laboratory settings and early clinical evaluations, as reported in multiple studies (Shaikh et al., 2020, Shaikh et al., 2022; Akhlaghpour et al., 2024; Mattoo and Spencer, 2024). However, in practice, their real-world implementation and application in routine clinical practice and their potential impact on AMR reduction still remains limited because of the persistent barriers for their implementation and uptake.

The major roadblocks for their implementation are policy and healthcare system inertia, majorly impacting their integration into standard clinical workflows, lack of infrastructure and training, cost incurred, and poor technology integration. Often, developers strive to build on decades of investment and harness the latest technologies to create useful PoCT tools. However, despite PoCTs catering to the clinical needs for quality healthcare, the uptake and implementation fail predominantly as a result of policy and decision makers failing to understand the value proposition of new technologies (Korte et al., 2020). For instance, Hermans et al (Hermans et al., 2024). in their 2024 PoCT in sub-Saharan Africa and low-resource settings review highlighted the failure of well-designed diagnostics deployment due to cost, lack of infrastructure, and poor health system integration. Further barriers in these regions are related to bias/prejudice of local healthcare personnel against new technologies, adherence to “traditional” diagnostic/treatment approaches, and lack of detailed, comprehensive, and targeted information campaigns that correlate the importance and impact of diagnostics and the direct relevance with treatment. A very crucial hinderance for adoption is also the cost of a diagnostic compared with that of an antibiotic. If the latter is cheaper, then, especially in low- and middle-income countries, the antibiotic is most likely to be administered than the diagnostic test to be done. This is why the cost of a diagnostic test should be competitive to that of antibiotics. A similar situation has been documented for PoCTs developed for other disease conditions as well (Jamshidi et al., 2024). There have been a number of PoCT platforms developed using new technologies that includes NAAT, biosensors, and rapid culture-based tools (Arienzo et al., 2016; Butler et al., 2018; Arienzo et al., 2023). **Table 1** and **Table 2** summarise the biosensors and PoCT platforms available for UTI that are commercially available, respectively. Yet, the adoption of these devices still remains a challenge. Perhaps, a modular, mix-and-match implementation strategy may enhance the effective deployment of rapid infectious disease and AMR PoCTs across transnational healthcare environments (Hays et al., 2019).

Similarly, in high-income countries, Korte et al (Korte et al., 2020). narrated that the adoption of PoCT often fails not due to poor test accuracy but because of misalignment between innovation goals and health economics and government rebates or reimbursement models, leading to resistance from different stakeholders including general practitioners (Korte et al., 2020).

Urine biomarkers play a crucial role in the development of PoCT for UTI, where biomarkers including neutrophil-associated lipocalin (NGAL), IL-8, and IL-1β have been shown to exhibit a positive correlation with symptomatic patients with microbial detection and multiplex PCR diagnosis (Akhlaghpour et al., 2024). In contrast biomarkers such as C-reactive protein and procalcitonin are not suitable or specific for distinguishing UTI and asymptomatic bacteriuria (Kuil et al., 2020, Kuil et al., 2021). In children, T-cell response proliferation markers, chemoattractants, and cytokines involved in interferon pathways have been identified to be promising in differentiating false positive results (Shaikh et al., 2020, Shaikh et al., 2022). NGAL and azurocidin (heparin binding protein) biomarkers have been identified as potential candidates for PoCT; however, their broad clinical significance is still in the research phase (Shaikh et al., 2020, Shaikh et al., 2022; Bilsen et al., 2024; Mattoo and Spencer, 2024).

In addition to biomarkers, optical technology and devices including colorimetric and fluorometric devices are promising for user-friendly PoCT for UTI diagnosis that are yet to be implemented in routine diagnosis, as reviewed elsewhere (Xu et al., 2023). Despite their proven value including Medicare Benefits Schedule (MBS) and the biomarker-supported PoCT platforms such as NGAL and IL-1β (Akhlaghpour et al., 2024; Bilsen et al., 2024), their widespread use in primary care as well as in emergency settings remains uncommon. Notably, a Dutch primary care projected that integration of PoCTs into clinical workflows would significantly reduce unnecessary prescriptions thus reducing the AMR risk (van der Pol et al., 2022).

Along with the above molecular platforms, Lateral Flow Tests (LFT) beyond the “traditional” antigen testing are being developed for analysing DNA, using isothermal amplification technologies (Brunauer et al., 2021). This is a very interesting approach as well as an easy-to-use PoCT that provides a higher level of information at the point of care, thereby assisting the decision making at primary care facilities. Eventually, a next generation of LFTs could be used to detect multiple analytes such as protein biomarkers and pathogen DNA (Klebes et al., 2022), as the combination of more than one class of biomolecules can provide a more accurate and complete diagnosis.

From an antimicrobial stewardship perspective, biomarkers that reliably differentiate symptomatic UTI from asymptomatic bacteriuria have direct implications for antibiotic prescribing decisions, particularly in high-burden settings such as RACFs and emergency departments where empirical treatment is common, as stated by several studies. Biomarker-guided approaches could support decisions to withhold or discontinue antibiotics in patients with non-specific symptoms or equivocal microbiological findings, thereby reducing unnecessary antimicrobial exposure. In acute care settings, early host-response biomarkers may also facilitate timely risk stratification and guided escalation or de-escalation of therapy. However, the translation of biomarker evidence into prescribing practice will depend on integration of the technology into point-of-care platforms, validation in real-world populations, and alignment with clinical workflows and stewardship protocols (Klebes et al., 2024).

## Emerging approaches in sample collection and logistics for rapid diagnostics

Novel approaches in sampling and logistics are emerging that include the home collection and dispatch in the form of dried urine spots (Suwanvecho et al., 2025). These are then, upon receipt by the analysing lab, rehydrated/reconstituted with dedicated extraction buffers, and the process of DNA extraction and purification begins, which will be followed by nucleic acid amplification (PCR or isothermal). This approach opens the door to population living in more remote, e.g., rural areas, to not need to be transported to large distances until the closest clinic/testing laboratory, especially if their condition is not yet acute. Furthermore, this approach promotes inclusivity and equity in healthcare as demonstrated during the COVID-19 era (Byström et al., 2023). Even more, given the fact that some patients with UTIs may be reluctant to visit the doctor, as they may be concerned to not be stigmatised, a self-collection and dispatch via dried urine spots may encourage them to be tested.

Along a similar line of “diagnostics goes to patients” instead of “patients go to diagnostics” is the concept of the “lab-in-a-suitcase”. It started and was first implemented in low- and middle-income countries in sub-Saharan Africa where access to diagnostics in rural areas is difficult (Faye et al., 2015; Weidmann et al., 2018), but nothing prevents such an option from being used in high-income countries as well (El Wahed et al., 2021), even in the big cities, as it is a fully customisable platform. Such an approach could significantly help decentralise healthcare and relieve the overloaded primary healthcare facilities, especially the GP offices. For elderly people and especially those with mobility issues, such approach could be ideal. It is frequently discussed about “home testing”, but the challenge there is always the self-sampling by the patient. However, as the lab-in-a-suitcase would be used by nursing personnel that visit the elderly homes for regular support (services like this already exist in Germany, e.g., Dreisam Sozialmedizinische Pflegebetriebe gGmbH Care with hand and heart), both the sampling and the test would be done at home by the nursing staff. Such a lab-in-a-suitcase would comprise standard tools that are required for analysis and, in combination with aforementioned rapid sample-to-result technologies, can provide the result whereas the nursing staff is still at the home of the patient. The results would then be sent to the GP who would make the final decision, e.g., whether the patient really needs to be transported to hospital, or can stay home with some recommendation for treatment through specific medication.

## Digitalisation and artificial intelligence for UTI diagnosis and combating AMR spread

Considering the present era being driven by digitalisation and heavy inclusion of artificial intelligence (AI) in the medical field, UTI has not been alienated. UTI datasets from patient records had been utilised to build digital models that are used in medical field such as decision tree (DT), support vector machine (SVM), random forest (RF), and artificial neural network (ANN). These were employed in the creation of definitive diagnosis for UTI and were found to be efficient with ANN holding the highest accuracy (Ozkan et al., 2018).

In the UK, heuristic model, ANN and extreme gradient boosting (EGB) were used and a heuristic model was implemented in routine clinical practice at a diagnostic pathology lab (Burton et al., 2019). Similarly, uroflowmetry data were used to create AI models to diagnose lower urinary tract dysfunction and urinary tract symptoms in men, which was found to be highly sensitive and specific (Matsukawa et al., 2021). Yet another study utilised DT, SVM, EGB, ANN, and RF to predict postoperative UTI in elderly women with ovarian cancer and found RF to be more helpful compared with others for treatment decision making (Ai et al., 2022). Furthermore, machine learning and AI have made significant positive impact in primary care sectors and in emergency care patients with UTI (Herter et al., 2022; Khan, 2022). In addition, AI has been employed for UTI diagnosis in several dimensions such as to improve antimicrobial stewardship, bacterial classification, urosepsis with upper urinary tract calculi, UTI with secondary blood stream infection, and catheter-associated UTI (Allugunti et al., 2023; Cai et al., 2023; Fadlil et al., 2023; Hong et al., 2023; Choi et al., 2024; Naik et al., 2024; Sufriyana et al., 2024).

Despite the growing number of AI-based models applied to UTI diagnosis and prediction, several limitations constrain their current clinical utility. Most published models rely on retrospective, single-centred datasets with internal validation only, raising concerns regarding robustness, overfitting, and generalisation across populations and healthcare settings. External validation using independent cohorts remains scarce; nevertheless, few studies assess model performance under real-world conditions but often lack true representation of different populations. In addition, algorithmic complexity, especially in ensemble and deep learning approaches, poses challenges in interpretation, which remains a critical barrier for clinicians to trust and adopt. Data bias arising from imbalanced cohorts, incomplete microbiological confirmation, and variability in diagnostic coding further complicate validation and model reliability. Consequently, most AI tools developed to date are best positioned as decision-support systems integrated into existing clinical workflows rather than being autonomous diagnostic solutions. Therefore, their impact on antimicrobial stewardship will depend on careful validation, transparency, and alignment with routine diagnostic pathways.

## Hurdles to diagnostic implementation

In summary, despite substantial advances in diagnostic technologies for UTIs, their translation into routine clinical practice remains limited due to persistent policy inertia, reimbursement challenges, and governance barriers in healthcare systems as discussed earlier. In many healthcare systems, reimbursement frameworks prioritise therapeutic interventions over diagnostics, creating a financial burden and disincentive for diagnostic testing despite the fact that such testing may reduce inappropriate antimicrobial use. The cost of a diagnostic test frequently exceeds empirical antibiotic therapy, especially in primary care and low-resource settings, unfortunately reinforcing empirical prescribing practices.

Healthcare system policy inertia further constrains adoption through fragmented healthcare pathways, scarce integration of diagnostics into clinical workflows, inadequate infrastructure, funding resources, time, and insufficient training. These constraints impact high-income settings as well due to misalignments between innovation timelines and regulatory approvals, reimbursement decisions, and guideline updates delays. As a consequence, diagnostics with strong analytical performance often fail to demonstrate real-world impact on prescribing behaviour and antimicrobial stewardship.

Collectively, all these discussed barriers highlight that diagnostic innovation alone is not adequate to mitigate UTI oriented AMR and/or AMR in general. Effective stewardship requires coordinated alignment between diagnostic development, healthcare governance/policy frameworks, reimbursement models, and clinical practice.

## Conclusion and future direction

UTIs remain a significant global health concern with implications for both individuals and healthcare systems causing both health and economic burdens. The diagnosis of UTIs has been challenging due to the lack of clarity leading to indiscriminate use of antibiotics, in turn leading to unintended consequences such as the disruption of the gut and urogenital microbiome and the development of AMR. The epidemiology of UTIs highlights the high prevalence among certain populations, including women, children, and residents of healthcare facilities. Timely and accurate diagnosis is crucial to avoid unguided antimicrobial treatment and unnecessary complications.

The management of UTIs and AMR requires a multifaceted approach and a careful balance between appropriate antibiotic use and minimising the emergence of AMR. Guidelines for treatment vary across different countries, leading to inconsistencies in empirical therapy and potential overuse of antimicrobials. The emergence of AMR, particularly among Gram-negative bacteria, raised and continues to raise concerns about the efficacy of traditional treatment options. It is imperative to explore and develop next-generation antimicrobials to address this challenging situation. That being said, improving the diagnosis of UTIs is crucial for guided treatment. While culture-based methods remain the gold standard, molecular tests, biosensors, host-specific biomarkers, and AI-mediated diagnosis show promise in providing rapid and accurate diagnoses. PoCT offers the potential for on-the-spot UTI diagnosis, reducing the turnaround time for results and enabling timely and targeted treatment. However, challenges around adoption and implementation always hinder their application in primary healthcare settings. For example, while AI-driven diagnostics show promise, their translation into routine UTI management will require rigorous external validation, attention to bias and interpretability, and integration as decision-support tools within existing diagnostic workflows. Their routine application in healthcare settings will take time; nevertheless, continued research and development in this field and awareness among healthcare workers including clinicians and specialists can enhance better understanding of UTIs, optimise treatment strategies, and facilitate personalised treatment approaches. In addition, a multidisciplinary approach involving clinicians, researchers, and policymakers is crucial to combat UTI and AMR effectively. This in turn will minimise the clinical and economic burden of UTIs and will facilitate better patient outcomes in the future.
